# Cytotoxicity of snake venom Lys49 PLA2-like myotoxin on rat cardiomyocytes ex vivo does not involve a direct action on the contractile apparatus

**DOI:** 10.1038/s41598-021-98594-5

**Published:** 2021-09-30

**Authors:** Alfredo Jesús López-Dávila, Natalie Weber, Theresia Kraft, Faramarz Matinmehr, Mariela Arias-Hidalgo, Julián Fernández, Bruno Lomonte, José María Gutiérrez

**Affiliations:** 1 Institute of Molecular and Cell Physiology, Hannover Medical School, 30625 Hannover, Germany; 2 Institute of Molecular and Translational Therapeutic Strategies, Hannover Medical School, 30625 Hannover, Germany; 3 Departamento de Fisiología, Escuela de Medicina, Universidad de Costa Rica, San José, 11501 Costa Rica; 4 Instituto Clodomiro Picado, Facultad de Microbiología, Universidad de Costa Rica, San José, 11501 Costa Rica

**Keywords:** Muscle contraction, Cell death, Myosin, Musculoskeletal models, Experimental models of disease

## Abstract

Viperid snake venoms contain a unique family of cytotoxic proteins, the Lys49 PLA_2_ homologs, which are devoid of enzymatic activity but disrupt the integrity of cell membranes. They are known to induce skeletal muscle damage and are therefore named ‘myotoxins’. Single intact and skinned (devoid of membranes and cytoplasm but with intact sarcomeric proteins) rat cardiomyocytes were used to analyze the cytotoxic action of a myotoxin, from the venom of *Bothrops asper*. The toxin induced rapid hypercontraction of intact cardiomyocytes, associated with an increase in the cytosolic concentration of calcium and with cell membrane disruption. Hypercontraction of intact cardiomyocytes was abrogated by the myosin inhibitor para-aminoblebbistatin (AmBleb). No toxin-induced changes of key parameters of force development were observed in skinned cardiomyocytes. Thus, although myosin is a key effector of the observed hypercontraction, a direct effect of the toxin on the sarcomeric proteins -including the actomyosin complex- is not part of the mechanism of cytotoxicity. Owing to the sensitivity of intact cardiomyocytes to the cytotoxic action of myotoxin, this ex vivo model is a valuable tool to explore in further detail the mechanism of action of this group of snake venom toxins.

## Introduction

Snake venoms contain a variety of cytotoxic components which induce local tissue damage in envenomings^1,2^. A family of cytotoxic proteins in venoms is comprised by phospholipases A_2_ (PLA_2_s) and by a group of PLA_2_-like homologs which lack catalytic activity owing to substitutions of key residues at the catalytic site and the calcium-binding loop. The most common variant of such catalytically-inactive PLA_2_ homologs is the so-called Lys49 PLA_2_s, characterized by having a Lys residue in substitution for the canonical Asp at position 49. In addition, these homologs present other substitutions in the calcium-binding loop^3,4^. Catalytically-active PLA_2_s exert cellular toxicity by enzymatic cleavage of phospholipids at the plasma membrane of a variety of cells, such as skeletal muscle fibers^5,6^, and nerve terminals in motor neurons^7^. In contrast, Lys49 PLA_2_ homologs are able to disrupt the integrity of the plasma membrane by mechanisms that do not rely on phospholipid hydrolysis^8,9^. Structure–function studies have identified a stretch of cationic and hydrophobic residues at the C-terminus of Lys49 PLA_2_ homologs as responsible for membrane disruption^10,11^. In addition, it has been proposed that these toxins have two independent interaction sites, for docking to and disruption of the plasma membrane^12^.

Studies on the mechanism of action of Lys49 PLA_2_ homologs have revealed their ability to interact with and disrupt natural and artificial membranes^13–18^. Such plasma membrane disruption leads to a prominent calcium influx, following an electrochemical gradient across the plasma membrane^17,19^. As a consequence, a series of intracellular degenerative events ensue, ending in irreversible cell damage^5,6,19^. Other mechanisms of cytotoxicity may be also involved, depending on concentration thresholds. For example, a Lys49 protein induced apoptosis in a lymphoblastoid cell line^16^. More recently, the possibility that these cytotoxins exert their action intracellularly after endocytosis has been raised, following the demonstration that a Lys49 PLA_2_ homolog binds to nucleolin at the plasma membrane and is then rapidly internalized and localizes in the nucleus and the perinuclear space in myogenic cell lines in culture^20^ and in muscle fibers in vivo^21^. Other studies have shown cytotoxicity in macrophages by the purinergic receptor-mediated action of ATP released from damaged cells^22^. Hence, Lys49 PLA_2_ homologs induce cellular toxicity by a variety of mechanisms that need to be further explored.

In contrast to the abundance of studies on cytotoxicity of skeletal muscle cells in vivo and in vitro, the action of these toxins on cardiomyocytes has received little attention. Cardiac muscle cells present similarities and differences with skeletal muscle fibers. Both are striated cells with similar contractile machineries. On the other hand, skeletal muscle fibers are multinucleated elongated cells, while cardiomyocytes have a short, branching pattern, being functionally integrated through intercalated discs^23^. Likewise, calcium homeostasis in these cells presents similarities and differences^24^. It is therefore of interest to analyze the action of Lys49 toxins on cardiomyocytes, in order to identify whether they are susceptible to their actions, and to understand the underlying mechanisms of toxicity.

In this study, the effects of a Lys49 PLA_2_ homolog on two ex vivo preparations of rat cardiomyocytes were assessed. Single intact cardiomyocytes were used to study direct cytotoxicity, disruption of plasma membrane, changes in intracellular calcium concentration and hypercontraction. In parallel, single skinned cardiomyocytes were used to investigate a direct effect of the toxin on the contractile apparatus (i.e., the sarcomeric proteins). Our findings demonstrate the cytotoxic action of a Lys49 PLA_2_ homolog on rat cardiomyocytes and provide evidence for a rapid disruption of plasma membrane integrity, associated with a prominent increment in cytosolic calcium and hypercontraction. Moreover, no direct effects on the contractile apparatus of cardiomyocytes were observed.

## Material and methods

### Experimental animals

All experiments were conducted in accordance to the German animal protection law and European Communities Council Directive 2010/63/EU for the protection of animals used for experimental purposes and were approved by the Institutional Animal Care and Research Advisory Committee and permitted by the local responsible authorities in Lower Saxony (LAVES, Oldenburg, Germany). Animals were housed according to the Federation of European Laboratory Animal Science Associations (FELASA) recommendations^25^. The study is reported in accordance with ARRIVE guidelines^26^. Cardiomyocytes isolated from adult male Lewis rats (250–300 g, Charles River, Germany) were used in all experiments.

### Myotoxin II

The Lys49 PLA_2_ homolog Myotoxin II (Mt-II) was isolated from the venom of adult specimens of the snake *Bothrops asper* collected in the Pacific versant of Costa Rica and maintained at the Serpentarium of Instituto Clodomiro Picado (University of Costa Rica). Isolation was carried out by ion-exchange chromatography on CM-Sheparose followed by reverse-phase (RP) HPLC chromatography, as previously described^18^. Homogeneity of the preparation was confirmed by RP-HPLC. The degree of purity of isolated Mt-II reached > 95% and was additionally confirmed by mass spectrometry. In order to make efficient use of Mt-II, toxin concentrations for the different assays were determined by means of preliminary experiments. At 50 µg/mL, Mt-II induced fast and strong effect on intact cardiomyocytes (time to hypercontraction, see below) while approaching the asymptote. Thus, this concentration was selected as the maximal meaningful one (further increases in concentration made only marginally faster effects) and was used for the experiments on skinned cardiomyocytes (preliminary experiments here suggested no effect of Mt-II). For the rest of the experiments, we selected 50% of the maximal concentration (i.e. 25 µg/mL) since it generates essentially the same effects that the maximal one while slowing down the process (longer time to hypercontraction, but on a reasonable timeframe). This not only saves toxin but also facilitates the observation and quantification of key phenomena (e.g. changes in intracellular calcium concentration).

### Solutions

Unless otherwise indicated, concentrations are given in mmol/L. For cardiomyocyte isolation: Krebs–Henseleit buffer (KHB): NaCl 118, KCl 4.7, NaHCO_3_ 21, MgSO_4_ 1.2, glucose 11, KH_2_PO_4_ 1.2, CaCl_2_ 1, taurine 5, 2,3-butanedione monoxime 10, pH 7.4, 37 °C. Ca^2+^-free KHB: same as KHB without added Ca^2+^. Supplemented DMEM: DMEM (E15810, PAA-Laboratories, Pasching, Austria) supplemented with 1% antibiotic solution (Pen/Strep, Biochrom AG, Berlin, Germany, 10,000 µg/mL). Supplemented M199: M199 (E15-834, PAA-Laboratories) supplemented with 10 mmol/L l-glutathione, 0.2 mg/mL bovine serum albumin and 1% antibiotic solution. For analysis of intracellular Ca^2+^ transients and sarcomere shortening and general effect of Mt-II on intact cardiomyocytes: HEPES solution: NaCl 117, KCl 5.7, NaH2PO4 1.2, MgSO4 0.66, glucose 10, sodium pyruvate 5, creatine 10, HEPES 20, CaCl2 1.25, pH 7.4, 37 °C. For mechanical experiments on skinned cardiomyocytes: Relaxation solution: NaATP 5.95, MgCl_2_ 6.04, EGTA 2.0, KCl 139.6, Imidazole 10, pH 7.0, 5 °C. Relaxing and activating solutions: Imidazole 10, MgCl_2_ 2.0, MgATP 1.0, EGTA (or CaEGTA) 1.0, sodium creatine phosphate 50, Creatine kinase 500 U/mL, pH 7.0, 8 °C. Solutions with different free Ca^2+^concentration (pCa) were obtained by mixing appropriate volumes of relaxing and activating solutions. For all solutions, chemicals were obtained from Sigma-Aldrich Chemie GmbH. (Munich, Germany), Merck Chemicals GmbH. (Darmstadt, Germany) and Fischer Scientific GmbH. (Schwerte, Germany). The myosin inhibitor para-aminoblebbistatin (AmBleb, see below) was obtained from Optopharma Ltd. (Budapest, Hungary).

### Cardiomyocyte isolation

Single ventricular cardiomyocytes were isolated under sterile conditions as described previously^27,28^. Briefly, rats were anesthetized with isoflurane (Baxter, Unterschleissheim, Germany) using a vaporizer (E-Z-systems Corp., Palmer, PA, USA) and anticoagulated with heparin sodium (1000 U) iv. A bilateral thoracotomy flap was created, the aortic arch was cannulated, and the cannula was fixed in the aorta. The heart was rapidly (30–45 s) excised and mounted on a pressure-constant Langendorff perfusion system (~ 52 mmHg, usually resulting in a perfusion flow of 10–15 mL/min). Retrograde coronary artery perfusion was initiated immediately after connecting the heart to the Langendorff apparatus with KHB equilibrated with carbogen (5% CO_2_, 95% O_2_) at 37 °C. After 5 min the perfusate was switched to Ca^2+^-free KHB and the heart was perfused for additional 3 min. Enzymatic digestion was initiated using collagenase type II (140 U/mL, Lot M8B10274 Worthington Biochemical Corp., Lakewood, NJ, USA) and hyaluronidase type I-S (52 U/mL, Lot 029K77001, Sigma–Aldrich, Taufkirchen, Germany) diluted in Ca^2+^-free KHB. After 10 min of perfusion, extracellular Ca^2+^ was increased in three steps to the end concentration of 1 mmol/L and the perfusion continued for additional 10 min. The heart was removed from the perfusion system, the ventricles were gently cut into pieces and resuspended in 10 mL of enzyme containing KHB. By mincing the tissue pieces using silanized glass pipettes pieces of digested myocardium were mechanically dissociated into single cardiomyocytes. The resulting cell suspension was washed twice by centrifugation (80 g, 1 min) and resuspended in KHB supplemented with 2% and then with 6% bovine serum albumin. The cardiomyocytes were centrifuged, resuspended in supplemented DMEM, and plated (24-well culture plates) onto sterile 18 mm glass coverslips precoated with 40 µg/mL natural mouse laminin (Invitrogen, Cat.: 23,017- 015, Carlsbad, CA, USA) at 1:9 ratio with supplemented M199. After a 2 h incubation period (37 °C, 5% CO_2_) to allow the cells to adhere, incubation medium was replaced with fresh supplemented M199, and cells were kept at 37 °C and 5% CO_2_ until microscopy or intracellular Ca^2+^ transient acquisition. Cardiomyocyte density and cell viability were determined using bright-field microscopy after every isolation. Myocytes with a rod like shape, clearly defined edges, and visible striations usually represent viable cells, whereas cells without these characteristics are non-viable. Structural integrity of cardiomyocytes after one typical isolation process was additionally examined by fluorescence microscopy after staining a key structural sarcomeric protein (α-myosin heavy chain) and the cell nucleus (DNA).

### General effect of Mt-II on intact cardiomyocytes

To evaluate the general effect of Mt-II on intact cardiomyocytes, plates containing HEPES solution and intact, adherent cells on coverslips were placed on an inverted microscope. In order to keep a suitable temperature, the surface of the inverted microscope holding the plates was previously set at 37 °C. Subsequently, pre warmed aliquots of HEPES solution containing increasing concentrations of Mt-II were gently added to the different wells, achieving increasing final concentrations of 12.5, 25, 37.5 and 50 µg/L Mt-II in the different wells. Four repetitions using cardiomyocytes from two different isolations were performed for every final concentration and bright field videos were recorded for later analysis. At every Mt-II concentration, the cardiomyocytes were exposed to the toxin until a final state was achieved (i.e. all viable cells showed hypercontraction, see below).

### Cardiomyocytes staining

Structural integrity after isolation. A fluorescent staining of the α-myosin heavy chain isoform (α-MyHC) of cardiomyocytes was performed as described previously^28,29^. Briefly, after fixation and permeabilization, cardiomyocytes were incubated for 1 h with rabbit anti-α-MyHC (rabbit-a-huMYH6, polyclonal, BioGenes, Berlin, Germany) primary antibody, rinsed with PBS without Ca^2+^/Mg^2+^ (L1825; BioChrom, Berlin, Germany) for 15 min, and incubated for another hour with a specific goat anti-rabbit secondary antibody Alexa Fluor 488 (polyclonal, A11008; Thermo Fisher, Dreieich, Germany). Nuclei were stained with DAPI according to the manufacturer´s instructions (ab228549; Abcam, Berlin, Germany). Fluorescent images were acquired using an Olympus IX51 microscope and processed with cellSens (Olympus Corp., Tokyo, Japan) and ImageJ software packages. Integrity of the cell membrane of intact cardiomyocytes after incubation with Mt-II and AmBleb. The apoptosis/necrosis assay kit (ab176749; Abcam, Berlin, Germany) was used according to the manufacturer's instructions in order to assess the integrity of cardiomyocytes´s cell membrane after Mt-II exposure. Briefly, fresh adherent (laminin-coated coverslips) intact cardiomyocytes were incubated with 25 µg/mL Mt-II for 40 min at 22 °C in the assay buffer. To avoid hypercontraction, 50 µM myosin inhibitor AmBleb was added shortly before Mt-II administration. After Mt-II/AmBleb incubation, assay buffer containing Mt-II and AmBleb was replaced with fresh one containing apoptosis/necrosis markers (Apoxin Deep Red Indicator and Nuclear Green DCS1, respectively) and cells were incubated for 40 min at 22 °C for staining. Finally, cells were fixed and fluorescence images were acquired as mentioned above. Control cells were exposed to the same protocol excepting Mt-II exposure.

### Analysis of intracellular Ca^2+^ transients and sarcomere shortening

Intracellular Ca^2+^ transients and sarcomere shortening of single intact cardiomyocytes were recorded simultaneously. Briefly, adherent cardiomyocytes were incubated for 30 min at 37 °C, 5% CO_2_ in supplemented M199 with 2 µM intracellular Ca^2+^ dye FURA-2 AM (InvitrogenTM Corp., Molecular probes, Eugene, OR, USA). Then, cells were rinsed twice for 15 min, and fluorescence and shortening measurements were acquired at 37 ± 0.5 °C in a custom-made chamber containing HEPES solution. For acquisition, cardiomyocytes were electrically stimulated at 1 Hz, 25 Volt, with MyoPacer EP Cell Stimulator. Intracellular Ca^2+^ transients were measured by recording the fluorescence emitted at 510 nm after excitation with alternating between wavelength of 340 and 380 nm (FURA-2 ratio) using a dual excitation fluorescence photomultiplier system and a variable-rate CCD video camera coupled to an inverted microscope^27–29^. Sarcomere length was recorded using Fast-Fourier Transform (FFT) analysis after region of interest in the cell with clear striations was defined. For experiments, 1 min baseline was recorded and then Mt-II containing HEPES solution was added to give 25 µg/mL Mt-II in the acquisition chamber. Ca^2+^ transients and sarcomere shortening (twitches) were continuously recorded until hypercontraction. For analysis, 10 single twitches with their respective Ca^2+^ transients corresponding to the last ten seconds of the baseline and the last ten seconds before hypercontraction were averaged in order to increase the signal to noise ratio and several parameters were calculated using IonWizard software (hardware and software of IonOptix Corp., Amsterdam, Netherlands).

### Mechanical experiments on skinned cardiomyocytes

Skinned cardiomyocytes lack cell membrane but conserve the full sarcomeric contractile machinery and can be firmly attached to a force transducer and a length driver. In this system the cytoplasmic solution is replaced by buffers of different Ca^2+^ concentrations and the corresponding contractile response of the cells is measured^30^. Skinned cardiomyocytes were prepared and mounted in the experimental set-up as described previously^31–34^. Briefly, single cardiomyocytes were mechanically isolated from flash-frozen ventricular tissue samples. Before mechanical isolation, tissue was defrosted in relaxation solution. During the isolation the tissue was kept on ice. To dissolve all membranous structures the myocytes were incubated for 5 min in relaxation solution containing 1% Triton X-100 (chemical permeabilization). To remove Triton, skinned cells were washed twice in relaxation solution and kept at 5° C up to 24 h before mechanical experiments were done. After chemical permeabilization both ends of the isolated cardiomyocytes were attached to a force transducer and a length driver such that force transients at different free Ca^2+^concentration (pCa) could be measured. Isometric force and the rate constant of isometric force redevelopment after a short period of isotonic shortening (*k*_TR_) were measured by cycling the cell between isometric steady state contraction and short periods of unloaded isotonic shortening with subsequent re-stretch to the initial (isometric) sarcomere length^35–37^. Maximum active isometric force (F_max_) at every pCa was obtained by subtracting passive force from the total force, i.e. F_max_ = F_total_ − F_passive_. To obtain the rate constants, *k*_TR_, force transients were fitted by a single-exponential function. Force–pCa-relations were fitted using a sigmoidal Hill equation.

$${F}_{n}\left(pCa\right)= \frac{1}{1+{10}^{{n}_{H}\left(pCa- {pCa}_{50}\right)}}$$

where F_n_ is the force at pCa =  − LOG_10_[Ca^2+^] normalized to maximum force at pCa 3.88, pCa_50_ is pCa when F_n_ = 0.5, and *n*H is the steepness (Hill coefficient) of the force − pCa relation. Tension development of skinned cardiomyocytes (kN/m^2^) was calculated based on their cross-sectional area. Mechanical experiments were performed at 8 °C. To minimize confounding factors, control and treatment (Mt-II) experiments were conducted alternating both kind of conditions. Staff performing and analyzing the experiments did not know the allocation. See Figure S1 in the supplementary information showing original data acquisition.

### Inhibition of force development by para-Aminoblebbistatin (AmBleb)

AmBleb is a derivative of blebbistatin^38,39^ that prevents myosin from entering the force-producing states and thus inhibits force development of striated muscle^36,37^. Because force development induces shortening, this treatment permits examination of the cardiomyocytes during and after Mt-II exposure while conserving their structure, since the observed Mt-II-induced hypercontraction is avoided. The concentration of AmBleb was determined using its extinction coefficient at 427 nm of 6100 M^−1^ cm^−1^ in DMSO^38^. The effective concentration of AmBleb (50 µM) required to inhibit most of the active force generation of cardiac myosin was determined previously^36^.

### Statistics and data analysis

All values are given as a mean ± SEM (standard error of the mean) with n representing the number of experiments. Mean values were compared using unpaired or paired Student’s *t*-test (*p* < 0.05) as appropriate. For the analysis of time to hypercontraction, mean values were compared using a one-way analysis of variance (ANOVA, *p* < 0.05) and post hoc analyses of Tukey (multiple comparison test for single effects). Statistical analysis was performed using GraphPad Prism (version 5.01) for Windows, GraphPad Software, San Diego, California USA, www.graphpad.com.

## Results

### Viability of harvested primary cardiomyocytes

**Figure 1:**
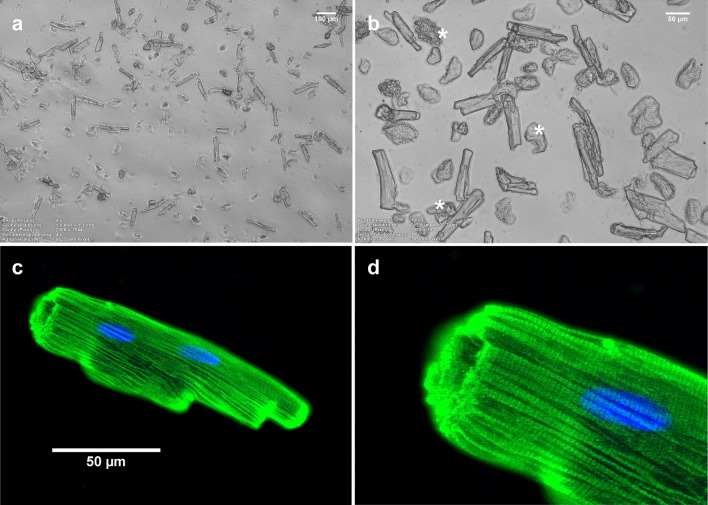
Primary cardiomyocytes harvested after a typical isolation process. (**a,b**) Bright-field microscopy showing a typical field of view on one coverslip with adhered cells in culture medium. In (**b**), as examples, three cells that do not meet the criteria to be considered viable are marked with an asterisk. (**c**) Fluorescence microscopy after α-MyHC immunostaining (green) and nuclear staining (blue, DAPI) confirmed structural integrity of a typical viable cardiomyocyte (adherent, rod like shaped and with clearly defined ends). (**d**) Magnification of the left-upper region of the cell showed in C. Note the characteristic striated pattern of cardiomyocytes. Images acquired and processed using Olympus cellSens (version 2.3) https://www.olympus-lifescience.com/de/software/cellsens/ and ImageJ (version 1.49.v) https://imagej.nih.gov/ij/ software packages.

Figure 1a,b show typical primary cardiomyocytes obtained after the isolation process. Viable cells are adherent, rod-like shaped and present clearly defined edges. Only viable cells were considered intact and were used for subsequent experiments. Cells lacking those traits were probably mechanically damaged during the isolation process and were used as positive controls in cell membrane staining experiments (see below). Figure 1c,d show the characteristic striated pattern of a representative intact cardiomyocyte, confirming its structural integrity.

### The general effect of Mt-II on intact cardiomyocytes is a permanent hypercontraction

**Figure 2:**
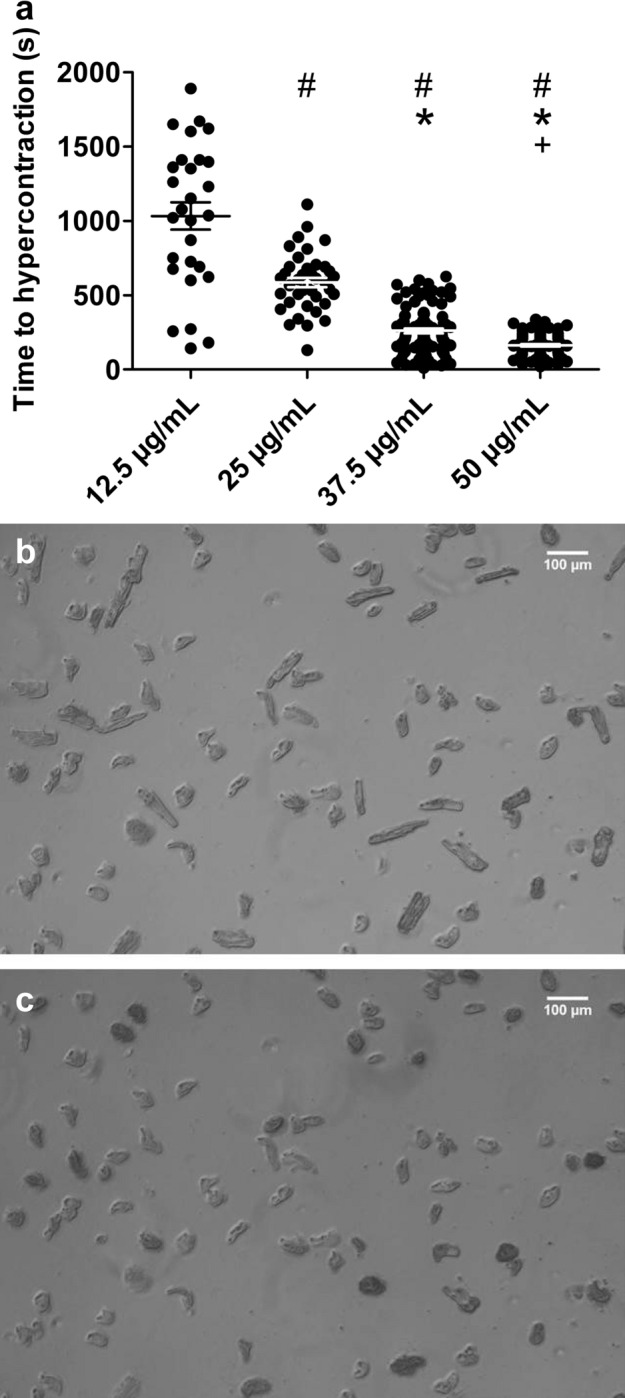
The general effect of Mt-II on intact cardiomyocytes is a permanent hypercontraction. (**a**) Intact cardiomyocytes were exposed to increasing Mt-II concentration in HEPES solution at 37 °C. A dose–response relation between Mt-II concentration and the time to reach hypercontraction was observed. Every dot represents a single cell. #, *, + : significantly shorter time to hypercontraction than at 12.5, 25 and 37.5 µg/mL, respectively (one-way ANOVA, *F* = 148.1, *p* < 0.001). Image created using GraphPad Prism (version 5.01) www.graphpad.com. (**b**,**c**) Bright-field microscopy images obtained during a typical experiment included in (**a**). The same region of a coverslip containing intact cells before (**b**) and after (**c**) exposure to 25 µg/mL Mt-II is shown. When exposed to Mt-II neighboring cardiomyocytes did not achieve hypercontraction simultaneously. After about 17 min of Mt-II exposure (**c**), however, all cardiomyocytes achieved hypercontraction. Supplementary videos 2 and 3 show original experiments of data included in a. Images acquired and processed using Olympus cellSens (version 2.3); https://www.olympus-lifescience.com/de/software/cellsens/

On intact cardiomyocytes, Mt-II induced permanent hypercontraction after a short period. The time needed to achieve the hypercontraction was inversely proportional to the concentration of toxin (17.3 min and 2.7 min for 12.5 and 50 µg/mL, respectively), describing a typical dose–response relation (Fig. 2a). Increasing Mt-II concentration not only significantly decreased the mean time for hypercontraction but also the standard deviation of the data. Supplementary video 1 shows the typical hypercontraction of intact cells after Mt-II exposure and supplementary videos 2 and 3 show typical experiments included in the data presented in Fig. 2. We selected two key steps of the excitation–contraction coupling of striated muscle in order to explain the observed hypercontraction: the contraction itself, given by the interaction of myosin with actin in the contractile apparatus of the cell (cross-bridges cycle, tested on skinned cardiomyocytes) and the electrically induced Ca^2+^ transient, which triggers contraction by activating the actin thin filament (tested on intact cardiomyocytes).

### Skinned cardiomyocytes are not affected by Mt-II

**Figure 3:**
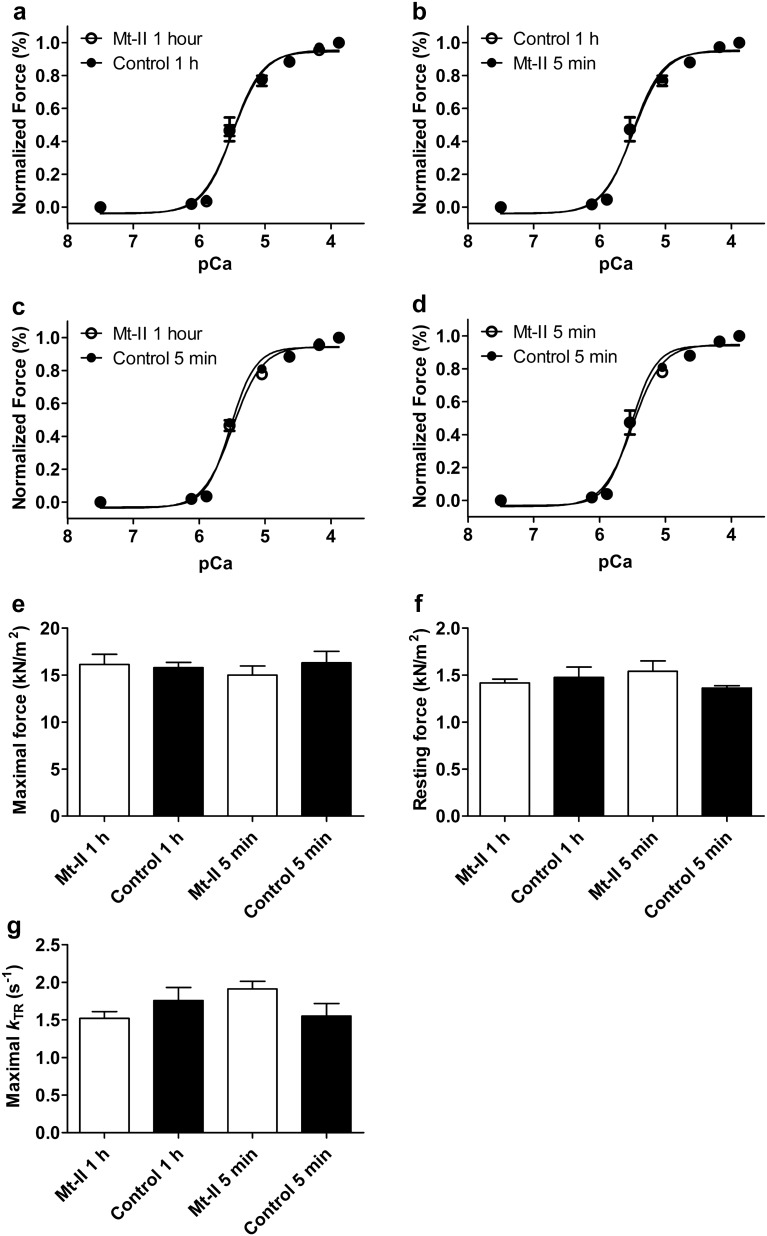
Skinned cardiomyocytes were not affected by Mt-II. (**a**) Force-pCa relation of skinned cardiomyocytes exposed to 50 µg/mL Mt-II for one hour in relaxing solution. Calcium-induced force development was not different from those of control cells undergoing the same procedure without Mt-II exposure. (**b**,**c**) A short time (5 min.) Mt-II exposure (same concentration as above) of control cells in (**a**) did not affect force pCa-relation (see **b**), which excludes the possibility of a short-term effect of Mt-II on a repeated measurements basis. A masked, accumulative effect in (**b**) (for example decreased force generation induced by mechanical cell deterioration with simultaneous increased force generation induced by the toxin) is excluded by data presented in (**c**), showing no mechanical cell deterioration in the second activation without toxin. (**d**) A short-term Mt-II effect is also excluded on an individual group basis, when short-term incubations of cardiomyocytes in (**b**) and (**c**) are compared. Data in (**d**) also confirm the high quality of skinned cardiomyocytes, since both groups of cells conserved similar patterns of force development in the second activation. (**e**) Maximal force (KN/m^2^) development at saturating calcium concentration (pCa 3.88) of control and Mt-II exposed cells shown in (**a**–**d**), including short-term (5 min) and long-term (60 min.) incubation experiments. There were no significant differences. (**f**) Same as in e for resting force at relaxing calcium concentration (pCa 7.50). (**g**) Rate constant of force development (*k*_TR_) at saturating calcium concentration (pCa 3.88). In spite of slightly higher standard deviations as for maximal and resting force, there were not significant differences between groups. For statistics, see Table 1. Note that figures (**a**) and (**b**) show two conditions each (control and Mt-II). Image created using GraphPad Prism (version 5.01) www.graphpad.com.

To evaluate short and long-term effects of Mt-II on the contractile apparatus, skinned cardiomyocytes were incubated with 50 µg/mL Mt-II for 5 min or 1 h. Cells undergoing the same procedures except for Mt-II exposure were used as controls. Those experiments are summarized in Fig. 3 and Table 1. Figure 3a shows the normalized force development of both skinned cardiomyocytes incubated with 50 µg/mL Mt-II in relaxing solution for one hour and control cardiomyocytes. This long-time exposure did not influence Ca^2+^ sensitivity nor cooperativity of force development, as evidenced by the similar pCa_50_ values and hill coefficients (n_H_) for both groups. To test for a possible immediate but transitory effect of Mt-II (thus not detectable after 60 min incubation) control cardiomyocytes were additionally incubated with 50 µg/mL Mt-II for 5 min after recording the data presented in Fig. 3a, and force development was recorded again (Fig. 3b). To control a possible effect in this experiment, cells exposed 1 h to Mt-II were additionally incubated in control conditions for 5 min and force development was also additionally acquired (Fig. 3c). Ca^2+^ sensitivity and cooperativity of force generation were not influenced by this short-term Mt-II incubation neither on a repeated measurement basis (Fig. 3b,c) nor on an independent group basis (Fig. 3d). pCa_50,_ n_H_ and force values of those experiments are shown in Table 1. Maximal and resting force (Fig. 3e,f, respectively) were not significantly different between those conditions, nor the rate constant of force redevelopment (*k*_TR_) at saturating Ca^2+^ concentration (Fig. 3g). Thus, Mt-II did not directly affect key parameters of force generation in skinned cardiomyocytes, in spite of its quick effect on intact cardiomyocytes.

**Table 1:** Force parameters of skinned cardiomyocytes.

	Control 1 h (n = 6)	Mt-II 1 h (n = 7)	Control 5 min. (n = 5)	Mt-II 5 min. (n = 5)
pCa_50_	5.45 ± 0.07	5.48 ± 0.03	5.52 ± 0.02	5.50 ± 0.06
n_H_	2.76 ± 0.68	2.38 ± 0.42	2.46 ± 0.32	2.92 ± 0.69
Maximal force	15.8 ± 0.57	16.1 ± 1.07	16.3 ± 1.22	15.0 ± 0.98
Resting force	1.47 ± 0.11	1.42 ± 0.04	1.36 ± 0,02	1.54 ± 0.11
Maximal *k*_TR_	1.76 ± 0.17	1.52 ± 0.09	1.55 ± 0.17	1.91 ± 0.10

pCa_50_: − LOG_10_[Ca^2+^] at 50% of maximum force generation. n_H_: Hill coefficient or slope of the force–pCa relation; denotes cooperativity of force development. *k*_TR_: rate constant of isometric force redevelopment after a short period of isotonic shortening. No significant differences (*p* > 0.05) were observed between control and Mt-II treated cardiomyocytes in any of the parameters tested.

### Mt-II induces an increase in intracellular Ca^2+^concentration and cell shortening in intact cardiomyocytes

**Figure 4:**
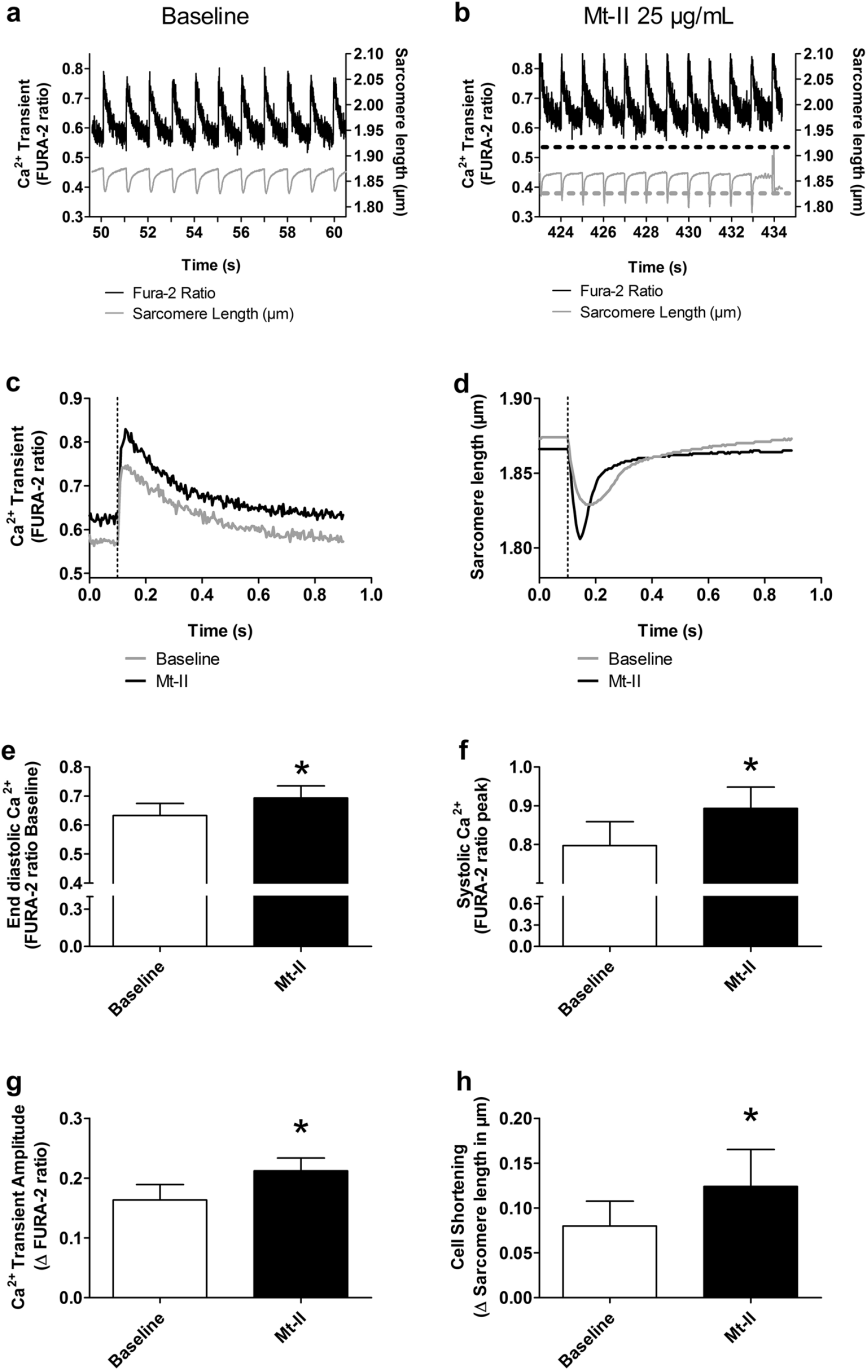
Mt-II induced a slow increase of intracellular Ca^2+^concentration and cell shortening in intact cardiomyocytes. (**a**) Intracellular calcium transients and sarcomere length of an intact cardiomyocyte during the last ten seconds of baseline phase, before Mt-II exposure (total baseline phase 60 s). (**b**) Same as in a after Mt-II exposure (25 µg/mL). The time interval 424–434 s post Mt-II exposure corresponds to the last ten seconds before hypercontraction was observed. The dotted lines represent baseline values shown in (**a**). Note the increased calcium concentration and corresponding increased cell shortening. (**c**) Averaged calcium transients of the ten second periods shown in (**a**) and (**b**). (**d**) Same as in c for sarcomere length. The vertical dotted lines in (**c**) and (**d**) show the timing of the electrical stimulus triggering the calcium transient and cell shortening. (**e**) Mean end diastolic calcium level (calculated before the electrical stimulus) of a group of cells undergoing the procedures as in (**a**–**d**) (control and Mt-II exposure). (**f**) Same as in (**e**) for systolic calcium level (the maximal value observed after the electrical stimulus) for the same two groups of cells. (**g**) Same as in (**e**–**f**) for amplitude of the calcium transient (difference between diastolic and systolic calcium). (**h**) Same as in (**e**–**g**) for mean cell shortening (difference between the longest and shortest sarcomere length registered before and after the electrical stimulus, respectively). Pacing rate in all experiments 1 Hz. *: statistically significant (*p* < 0.05). See statistics in Table 2. Image created using GraphPad Prism (version 5.01) www.graphpad.com.

**Figure 5:**
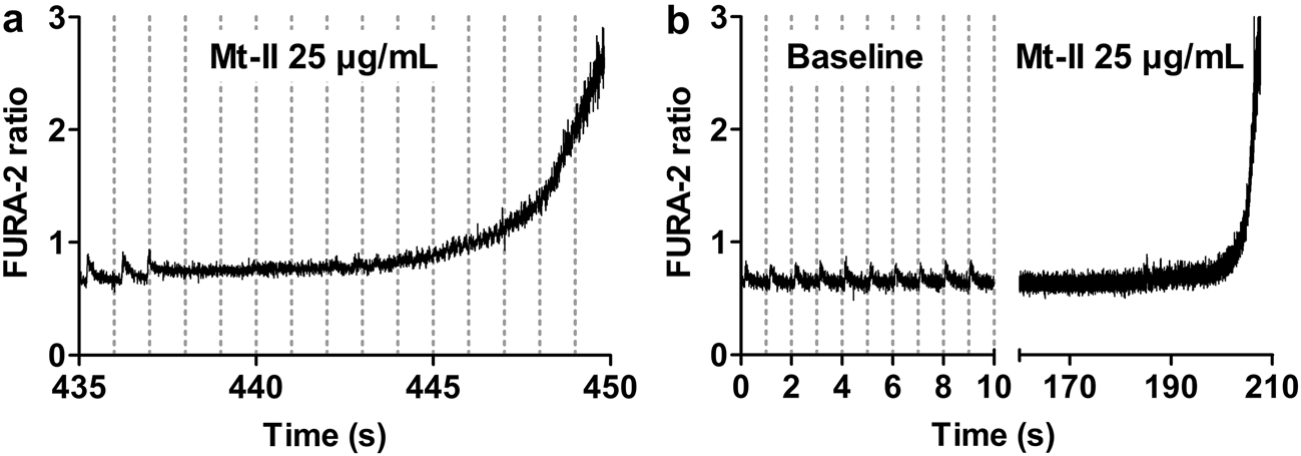
Mt-II induced cell hypercontraction is characterized by a massive increase of intracellular calcium concentration, regardless of presence or absence of electrical cell stimulation. Vertical dotted lines in a and b show the timing of electrical stimuli. (**a**) Calcium transients of the cell presented in Fig. 4a,b after 434 s of Mt-II exposure. Striation pattern was lost at 434 s, making sarcomere length acquisition no longer possible. Few calcium transients were still registered before the cell became stagnant to the electrical stimulus for several seconds. Note also the delayed calcium transient at 435 and 436 s. Finally, a massive increase of intracellular calcium was registered, which occurred simultaneously with the hypercontraction of the cell. (**b**) Intracellular calcium concentration of an intact cardiomyocyte exposed to Mt-II in absence of electrical stimulation. The left segment of the X-axis corresponds to the first ten seconds of the baseline phase (total baseline time 60 s). Electrical stimuli were applied only during the first ten seconds in order to confirm cell responsiveness. Thereafter, the electrical pacer was turned off and FURA-2 signal acquisition was continued. The right segment of the X-axis corresponds to Mt-II exposure, after 1 min baseline. There were no calcium transients because an electrical stimulation was not administrated. Again, a massive increase of intracellular calcium at time of hypercontraction was observed, as in presence of electrical stimulation in (**a**). The apparently different slopes of data in (**a**,**b**) obey mostly to the different time scales used. All cells exposed to these conditions presented the same behavior, including some variation of the time interval until hypercontraction at a given Mt-II concentration, as shown in Fig. 2a. Image created using GraphPad Prism (version 5.01) www.graphpad.com.

Figure 4a shows a representative time course of intracellular Ca^2+^concentration measured by FURA-2 ratio and sarcomere length of an intact cardiomyocyte before exposure to Mt-II (baseline). After 1 min baseline acquisition, 25 µg/mL Mt-II were added to the HEPES solution. After Mt-II addition, the intracellular Ca^2+^concentration and magnitude of cell shortening of the same cardiomyocyte slowly increased (Fig. 4b). Figure 4c,d respectively show the mean Ca^2+^transient and the mean sarcomere length (average of 10 cycles) of the same cell before and after Mt-II exposure. Several parameters were analyzed and compared from these averaged recordings. The end diastolic Ca^2+^concentration (registered before the electrical stimulus; indicated as dotted line in Fig. 4c,d) was significantly increased after Mt-II treatment (Fig. 4e). The systolic Ca^2+^concentration (maximal concentration registered after the electrical stimulus) as well as the total transient amplitude (Fig. 4f,g, respectively) were also significantly increased by Mt-II. The total amplitude of the sarcomere length change was accordingly increased (Fig. 4h). All values are included in Table 2. Note that experiments presented in Fig. 4 are essentially the same as those presented in Fig. 2, with the additional acquisition of intracellular Ca^2+^concentration and sarcomere length. Thus, cells presented in Fig. 4 show hypercontraction after few minutes, which is characterized by a massive increase in the intracellular Ca^2+^concentration and the loss of the striations (and consequently, the abrupt interruption of the sarcomere length recording, see the last cycle of Fig. 4b). Figure 5 presents the typical increase in intracellular Ca^2+^ at hypercontraction, which was preceded by delays between the application of the electrical stimulus and the onset of the Ca^2+^ transient (Fig. 5a). A separated group of cells was exposed to Mt-II in the absence of electrical stimulation. In these cells, the same increase in intracellular Ca^2+^ at hypercontraction was observed. Figure 5b shows a representative experiment illustrating the typical response of these cells. Altogether, data of skinned and intact cardiomyocytes suggested that Mt-II induced hypercontraction was the result of increased intracellular Ca^2+^ concentration without a direct effect on the contractile apparatus, i.e., thin and thick filaments.

**Table 2:** Calcium transients and associated cell shortening in viable cardiomyocytes.

	Baseline	Mt-II	*P* Value	N
Diastolic Ca^2+^	0.63 ± 0.04	0.69 ± 0.04	0.0138	6
Systolic Ca^2+^	0.79 ± 0.06	0.89 ± 0.05	0.0174	6
Ca^2+^ Transient amplitude	0.16 ± 0.02	0.21 ± 0.02	0.0322	6
Shortening	0.08 ± 0.03	0.12 ± 0.04	0.0398	6

### The myosin inhibitor AmBleb precludes hypercontraction of intact cardiomyocytes exposed to Mt-II

**Figure 6:**
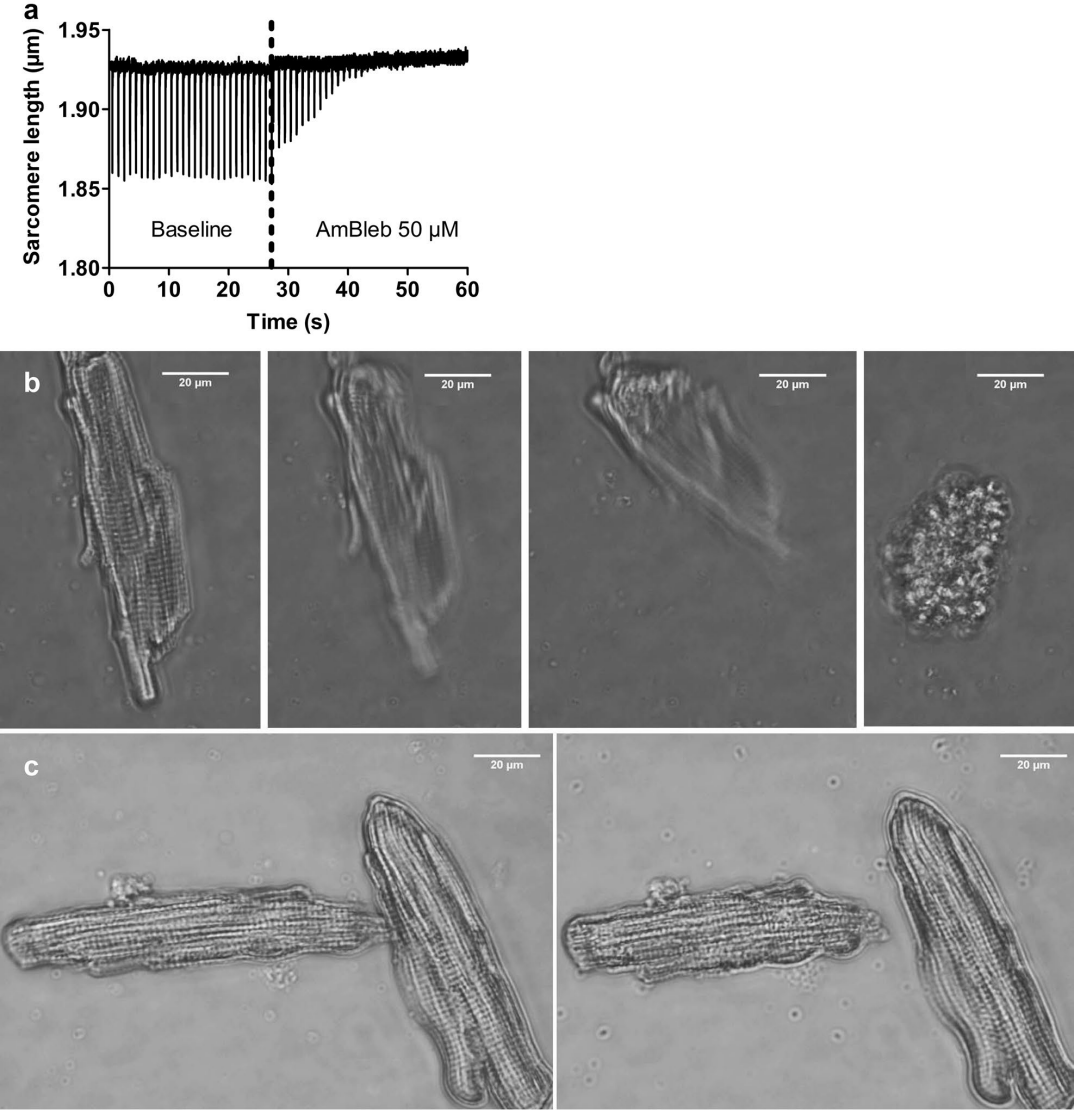
The myosin inhibitor AmBleb abrogated hypercontraction of intact cardiomyocytes exposed to Mt-II. (**a**) Electrically induced (1 Hz) shortening of a viable cardiomyocyte (baseline). The experiment is essentially the same as that depicted in Fig. 4a, but before FURA-2 loading, so that only sarcomere length can be acquired. The dotted line shows the timing of administration of a bolus of HEPES solution containing AmBleb, yielding an AmBleb concentration of 50 µM in the chamber (the small drift of the signal is an artifact resulting from the bolus administration). In less than 20 s, the cell becomes stagnant to the electrical stimulus. Image created using GraphPad Prism (version 5.01) www.graphpad.com. (**b**) Bright field microscopy of an intact cardiomyocyte shortly after exposure to 50 µg/mL Mt-II. Note the strong, irreversible shortening, i.e., hypercontraction. The sequence of pictures was obtained from the supplementary video 4. (**c**) Bright-field microscopy of two intact cardiomyocytes shortly after exposure to 50 µg/mL Mt-II, this time in presence of 50 µM AmBleb. In spite of a partial shortening (right picture in **c**), the difference with the end state in (**b**) is substantial. The sequence of pictures was obtained from the supplementary video 5 (see Fig. S2 in the supplementary information for additional photos at lower magnification). Experiments shown in (**b**,**c**) were performed in HEPES solution. Images (**b**) and (**c**) acquired and processed using Olympus cellSens (version 2.3); https://www.olympus-lifescience.com/de/software/cellsens/

Muscle shortening (concentric contraction) is essentially induced by actomyosin interaction, as described by the cross-bridge theory of striated muscle^40–42^). Thus, we hypothesized that treatment with the myosin inhibitor AmBleb -by preventing the cross-bridges from entering the strong binding states- would preclude Mt-II induced hypercontraction, even though the direct trigger of Mt-II-induced hypercontraction is not myosin itself but the increased intracellular Ca^2+^concentration. Figure 6a shows the fast effect of AmBleb on an intact cardiomyocyte, as evidenced by the fast interruption of cell shortening, despite continuous electrical stimulation. Figure 6b and supplementary video 4 show the typical hypercontraction of a single intact cardiomyocyte exposed to 50 µg/mL Mt-II. As evidenced by Fig. 6c, Fig. S2 in the supplementary information and supplementary video 5, treatment with AmBleb before Mt-II exposure largely precluded hypercontraction. In those cells, the intracellular Ca^2+^ concentration increased after Mt-II exposure (as evidenced by the partial shortening, see Fig. 6c), but the last effector of the hypercontraction (myosin) was strongly inhibited.

### Mt-II disrupts the cell membrane integrity of viable cardiomyocytes

**Figure 7:**
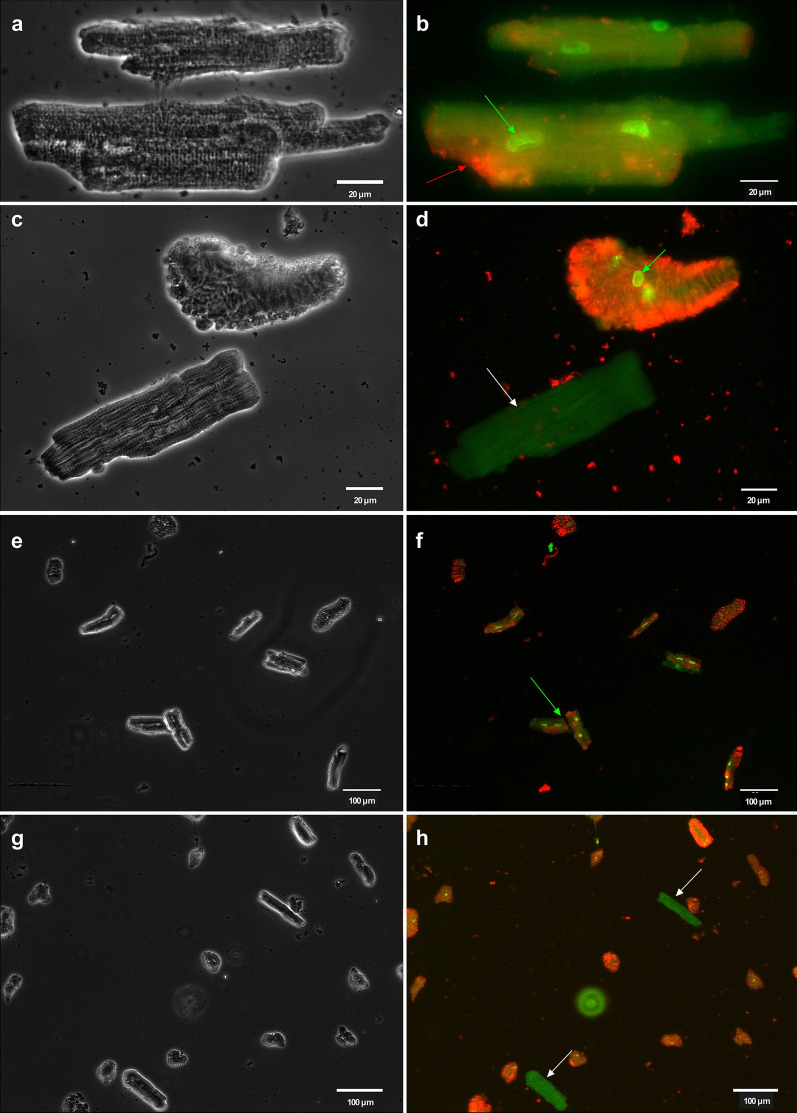
Mt-II disrupted the cell membrane integrity of intact cardiomyocytes. (**a,b**) respectively show bright field and fluorescence microscopy of two intact cardiomyocytes exposed to 25 µg/mL Mt-II (40 min). The nuclei of these intact cardiomyocytes were clearly stained after Mt-II exposure (see the green arrow in **b** showing one stained nucleus). In order to become permeable for the nuclear dye (DNA nuclear green DCS1), disruption of the cell membrane is required. Note that Apoxin Deep Red—a marker for phosphatidylserine—also stained some regions of the cell membrane (see the red arrow in **b** showing one of several stained regions). (**c,d**) respectively show bright field and fluorescence microscopy of two control cardiomyocytes exposed to the nuclear and membrane dyes without previous Mt-II treatment. The cell membrane of the upper cell was probably mechanically damaged during the isolation process. Note that it does not meet the criteria to be considered viable. Note also the green arrow in (**d**) showing that the nuclei were stained, as well as the strong red staining showing phosphatidylserine. On the contrary, the nucleus and cell membrane of the lower cell in (**d**) (identified by a white arrow) were not stained, suggesting that the membrane was not disrupted in this intact cell in absence of Mt-II. This control cell presented solely a low auto-emission in a similar spectral region of DNA nuclear green DCS1. (**e–h**) show the same scenarios as in (**a**–**d**) at lower magnification. (**e**,**f**) include Mt-II treated cells (thus comparable with **a**,**b**) while g and h include control cells (comparable with **c**,**d**). The green arrow in (**f**) shows two of six cells showing nuclei and phosphatidylserine staining after Mt-II treatment. The rest of the cells in (**f**) cannot be considered viable. White arrows in (**h**) show two control cells showing no staining but only some auto-emission as in (**d**). The rest of the cells in (**h**) cannot be considered viable and were affected either during the isolation process or during the several solution exchanges in order to apply the nuclear and membrane markers, since they were not exposed to Mt-II. Images acquired and processed using Olympus cellSens (version 2.3) https://www.olympus-lifescience.com/de/software/cellsens/ and ImageJ (version 1.49.v) https://imagej.nih.gov/ij/ software packages.

Avoiding hypercontraction with AmBleb as shown in Fig. 6c allows easy identification of key cellular structures after staining the Mt-II exposed cardiomyocytes. Figure 7a (bright field) and 7b (fluorescence) show intact cardiomyocytes stained after exposure to 25 µg/mL Mt-II for 40 min. Only intact cell membranes are impermeable to the green dye (DNA nuclear green DCS1). Thus, the strongly stained cell nuclei in Fig. 7b reveal disruption of the cell membrane, as in cell necrosis. Membranes of control cells unexposed to Mt-II remained impermeable to the nuclear green dye (Fig. 7c,d, showing bright field and fluorescence microscopy, respectively) as evidenced by the absent staining of the nuclei. Apoxin Deep Red, a phosphatidylserine dye, also stained cell membranes of cardiomyocytes exposed to Mt-II (Fig. 7b), but not of control cells (Fig. 7d). Figure 7e–h show control and Mt-II treated intact cardiomyocytes at lower magnification.

## Discussion

Acute skeletal muscle damage, i.e., myonecrosis, is a common clinical finding in viperid snakebite envenomings, often leading to sequelae due to poor muscle regeneration^2,43^. Venom-induced myotoxicity is mostly due to the action of PLA_2_s or PLA_2_ homologs^5,8^. There is a large body of information on the mechanism of action of these myotoxins on skeletal muscle cells (see reviews by Gutiérrez and Ownby^5^, Montecucco et al.^6^, and Lomonte and Rangel^8^). In contrast, few studies have analyzed the action of venom PLA_2_s and PLA_2_ homologs on cardiac muscle. Despite the fact that, with few exceptions, cardiotoxicity is not a common finding in snakebite envenoming^1,2^, the study of the effects of myotoxins on myocardial cells is of interest to further understand the mechanisms of cytotoxicity of venom components in striated muscle.

Previous studies have addressed the action of venom PLA_2_s in cardiac muscle ex vivo. A PLA_2_ from the venom of *Naja nigricollis* induced cardiotoxicity on isolated rat ventricular wall^44^ and on isolated perfused rat hearts^45^. Cardiotoxicity was also described in an isolated perfused rat heart preparation by the action of a PLA_2_ homolog from *Vipera ammodytes* venom. This toxin caused cardiac arrest, myocardial contracture and increase in intracellular markers in the sinus effluent^46^. We have studied the action of a Lys49 PLA_2_ homolog on isolated cardiomyocytes obtained from rats. The method of cell isolation described allows the analysis of cellular events in single cells, thus opening the possibility of assessing in a finer detail the mechanisms of cytotoxicity involved. The actions of myotoxins have been studied in skeletal muscle myotubes in culture^15,17,19,47–50^, but to the best of our knowledge this is the first report of the action of a venom toxin on single cardiomyocytes, as well as the first one dissecting its effects on intact and skinned cells.

Mt-II caused a rapid hypercontraction of cardiomyocytes, whose time of onset was dose-dependent. Hypercontraction has been widely described as an early change in skeletal muscle after administration of myotoxic PLA_2_s and PLA_2_ homologs in vivo^10,51,52^ and in myotubes in cell culture^17^. It has been proposed that such phenomenon in skeletal muscle is due to the calcium influx resultant from the myotoxin-induced disruption in the integrity of skeletal muscle plasma membrane^17,19,51^, although a rapid and transient elevation of cytosolic calcium from intracellular stores in myoblasts and myotubes was described before a massive influx through damaged plasma membrane^19^.

In the case of cardiomyocytes, a rapid increment in intracellular calcium was observed, which correlated with a reduction in the sarcomere length and with hypercontraction. This increment was observed for both end diastolic and systolic calcium levels and occurred similarly in the presence and absence of electrical stimulation, suggesting that it does not derive from calcium mobilization secondary to electrical stimulus. Moreover, a concomitant loss in the integrity of the plasma membrane was observed as judged by the intracellular staining by nuclear green. Thus, our observations suggest that Mt-II directly alters the integrity of the plasma membrane, inducing a rapid calcium influx from the extracellular space which causes hypercontraction and irreversible cell damage, similarly to what has been described for the action of these toxins in skeletal myotubes in culture^17,19^. Since Mt-II is devoid of catalytic activity and does not hydrolyze membrane phospholipids in cells^9^, the membrane disruption is due to phospholipid bilayer alterations not related to enzymatic cleavage. It remains to be investigated whether other organelles involved in cellular calcium homeostasis, i.e., sarcoplasmic reticulum and mitochondria, may also contribute to this calcium increase. Interestingly, cardiomyocytes affected by Mt-II showed staining with the phosphatidylserine dye Apoxi Deep Red. Since this phospholipid is predominantly present in the inner leaflet of the plasma membrane, in agreement with the lack of staining in control cells, this is another evidence of plasma membrane disruption, with exposure of this phospholipid to this dye as a consequence of such disruption. Alternatively, this staining may reveal the onset of apoptosis in cardiomyocytes. Further studies are needed to discern between these explanations.

Owing to the prominent hypercontraction induced by Mt-II, we assessed whether the toxin has any direct effect on the contractile apparatus of cardiomyocytes. To this end we used skinned cardiomyocytes which lack key membranous structures (plasma membrane, sarcoplasmic reticulum, and mitochondria) but have an intact contractile machinery^30,31^, such that contractile properties of sarcomeric proteins can be measured under standardized conditions, without other factors operating in intact cells (i.e., Mt-II induced elevation of cytoplasmic calcium). No differences were observed in various contractile parameters between skinned cardiomyocytes in the presence or absence of Mt-II. This indicates that the toxin does not exert a direct effect on the contraction mechanisms of myofibrils, and that the hypercontraction is the result of the cytosolic calcium increase and consequent activation of the thin filament and cross-bridges cycle, secondary to plasma membrane alteration.

The myosin inhibitor AmBleb abrogated Mt-II-induced hypercontraction in cardiomyocytes. This inhibitor is a valuable tool to further explore the mechanism of action of Mt-II since, by avoiding hypercontraction, it allows the observation of changes occurring in other organelles that may be affected by the action of this toxin, such as mitochondria, sarcoplasmic reticulum, lysosomes, or nuclei. In the case of skeletal muscle, Mt-II binds to nucleolin, a nucleolar protein that is also located in the plasma membrane^20^. It has been shown that such binding plays a role in the action of Mt-II. Moreover, this myotoxin has been located in the nuclei of skeletal muscle cells in vitro^20^ and in vivo^21^. It will be of interest to assess whether a similar process of internalization and localization in the nuclei also occurs in cardiomyocytes, a phenomenon that could be studied in cells incubated with the myosin inhibitor to better visualize intracellular structures. Moreover, AmBleb could become a valuable tool to analyze whether cellular hypercontraction contributes to further cellular damage by mechanically disrupting the integrity of cellular membranes, an issue that deserves further investigation.

In conclusion, an experimental model combining isolated rat cardiomyocytes and a myosin inhibitor has been developed to investigate the mechanism of action of venom myotoxins. This model allows the collection of consistent and reproducible results and the assessment of diverse cellular processes involved in cytotoxicity, including alterations in calcium homeostasis, plasma membrane disruption, changes in the contractile apparatus, and also alterations in other cellular organelles and cellular events. Our observations showed that Mt-II, a Lys49 PLA_2_-like homolog, induces rapid plasma membrane disruption, calcium influx and increments in cytosolic calcium concentration leading to hypercontraction, without directly affecting the contractile machinery of the cell.

## Supplementary Information

Supplementary Video 1.

Supplementary Video 2.

Supplementary Video 3.

Supplementary Video 4.

Supplementary Video 5.

Supplementary Information 1.

Supplementary Information 2.

## Data Availability

The datasets generated during and/or analyzed during the current study are available from the corresponding author on reasonable request.

## Abbreviations

AmBleb: Para-aminoblebbistatin
PLA_2_: Phospholipase A_2_
Lys49 PLA_2_: PLA_2_ homologs characterized by having a Lys residue in substitution for the canonical Asp at position 49
Mt-II: Lys49 PLA_2_ homolog Myotoxin II
*k*_TR_: The rate constant of isometric force redevelopment after a short period of isotonic shortening
pCa: − LOG_10_[Ca^2^^+^] (see Hill equation in the methods section)
*n*H: Hill coefficient (see Hill equation in the methods section)
