# Supplementary material for: Cytotoxicity of snake venom Lys49 PLA2-like myotoxin on rat cardiomyocytes ex vivo does not involve a direct action on the contractile apparatus
Source: Sci Rep. 2021 Sep 30;11:19452. doi: 10.1038/s41598-021-98594-5 (PMC8484475; doi:10.1038/s41598-021-98594-5)
Supplement: Supplementary file 6 — Supplementary Information 1. [file 41598_2021_98594_MOESM6_ESM.pdf]

Supplementary Videos

Cytotoxicity of snake venom Lys49 PLA2-like myotoxin on rat cardiomyocytes ex vivo does not involve a direct action on the contractile apparatus

Alfredo Jesús López-Dávila [1*], Natalie Weber[1,2], Theresia Kraft[1], Faramarz Matinmehr[1], Mariela Arias-Hidalgo[3], Julián Fernández[4], Bruno Lomonte[4], José María Gutiérrez[4]

1. Institute of Molecular and Cell Physiology, Hannover Medical School, Hannover 30625, Germany
2. Institute of Molecular and Translational Therapeutic Strategies, Hannover Medical School, Hannover 30625, Germany
3. Departamento de Fisiología, Escuela de Medicina, Universidad de Costa Rica, San José 11501, Costa Rica
4. Instituto Clodomiro Picado, Facultad de Microbiología, Universidad de Costa Rica, San José 11501, Costa Rica

*corresponding author. E-Mail address: lopezdavilacr@yahoo.es

Supplementary video 1. Mt-II induces strong and irreversible hypercontraction of intact cardiomyocites. After a short baseline period, a bolus of Mt-II-containing HEPES solution yielding a Mt-II concentration of 50 µg/mL was gently administrated in a well containing intact cells on a coverslip. Note the asynchronous reaction of neighboring cells to Mt-II with some of them twitching strongly while others are barely affected. After a longer time span, however, all cells achieve the same final state of irreversible hypercontraction. Bright-field microscopy. Magnification: 20X. Temperature 37°C.

Supplementary video 2. Example of acquisition of data presented in Fig. 2a. A bolus of Mt-II-containing HEPES solution yielding a Mt-II concentration of 25 µg/mL was gently administrated in a well containing intact cells on a coverslip. The same region of the coverslip was continuously observed. At this Mt-II concentration, the response of the cells was slow and the final state of hypercontraction was typically achieved after 15-20 min as shown in Fig. 2c. Bright-field microscopy. Magnification 4X. Temperature 37°C.

Supplementary video 3. Example of acquisition of data presented in Fig. 2a. Same conditions as in Online Resource 2 but at 50 µg/mL Mt-II. Note the faster, stronger response of the cells to this toxin concentration.

Supplementary video 4. Hypercontraction of an intact cardiomyocyte shortly after exposure to 50 µg/mL Mt-II in HEPES solution. Bright-field microscopy. Magnification 40X. Temperature 37°C.

Supplementary video 5. Same conditions as in Online Resource 4 but in presence of 50 µM AmBleb. Note that the myosin inhibitor precludes the Mt-II induced hypercontraction of the cells.
