# Supplementary material for: Cytotoxicity of snake venom Lys49 PLA2-like myotoxin on rat cardiomyocytes ex vivo does not involve a direct action on the contractile apparatus
Source: Sci Rep. 2021 Sep 30;11:19452. doi: 10.1038/s41598-021-98594-5 (PMC8484475; doi:10.1038/s41598-021-98594-5)
Supplement: Supplementary file 7 — Supplementary Information 2. [file 41598_2021_98594_MOESM7_ESM.pdf]

Supplementary Information

Cytotoxicity of snake venom Lys49 PLA2-like myotoxin on rat cardiomyocytes ex vivo does not involve a direct action on the contractile apparatus

Alfredo Jesús López-Dávila [1*], Natalie Weber[1,2], Theresia Kraft[1], Faramarz Matinmehr[1], Mariela Arias-Hidalgo[3], Julián Fernández[4], Bruno Lomonte[4], José María Gutiérrez[4]

1. Institute of Molecular and Cell Physiology, Hannover Medical School, Hannover 30625, Germany
2. Institute of Molecular and Translational Therapeutic Strategies, Hannover Medical School, Hannover 30625, Germany
3. Departamento de Fisiología, Escuela de Medicina, Universidad de Costa Rica, San José 11501, Costa Rica
4. Instituto Clodomiro Picado, Facultad de Microbiología, Universidad de Costa Rica, San José 11501, Costa Rica

*corresponding author. E-Mail address: lopezdavilacr@yahoo.es

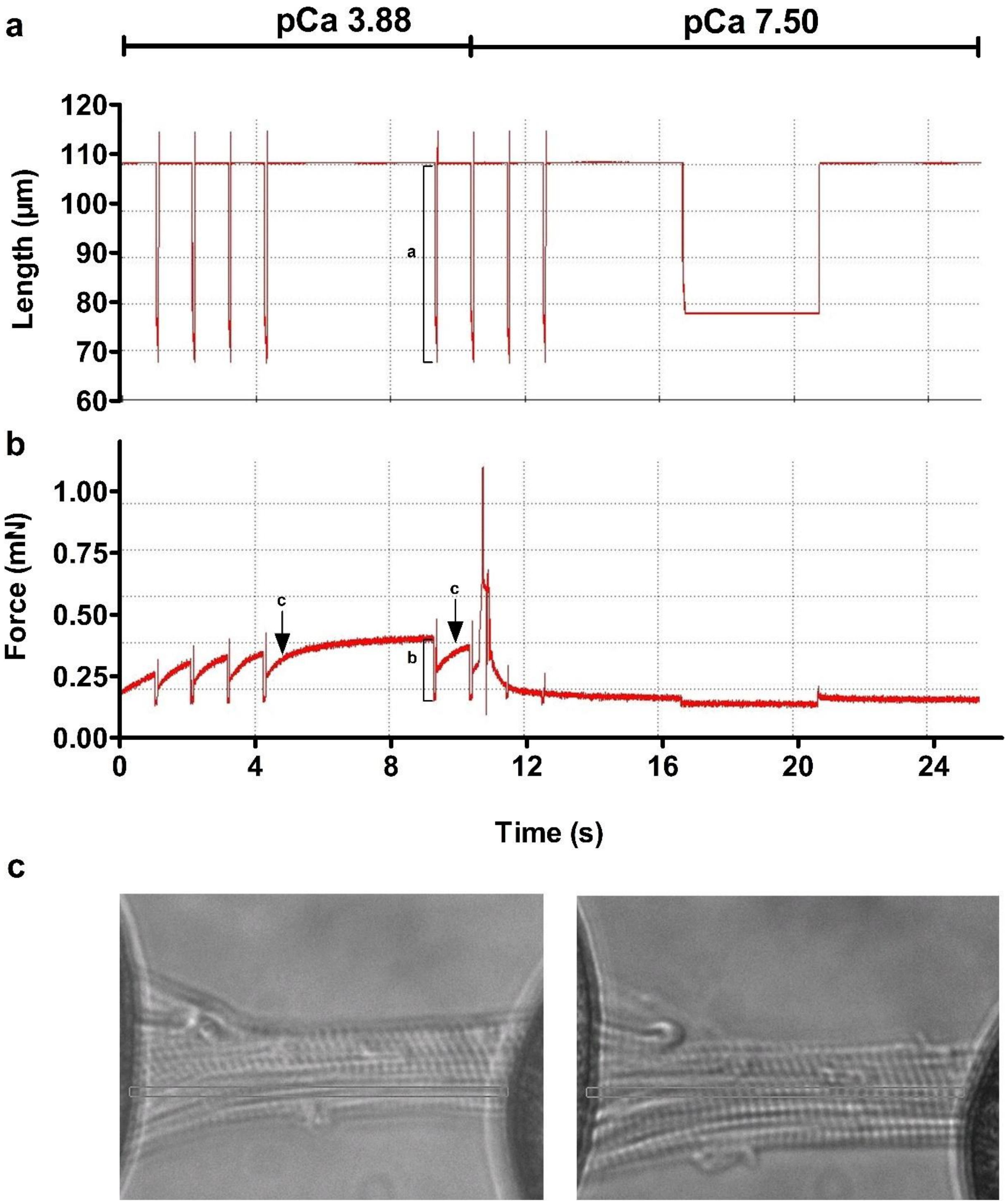

Fig. S1 Original experiment showing acquisition of maximal and resting force and maximal k_{TR}. **a and b** An isometrically held skinned cardiomyocyte was placed in a chamber containing activating solution (saturating calcium concentration; pCa 3.88). After several cycles of cell release-re-stretch (shown in a) allowing the acquisition of maximal force and maximal k_{TR} (shown in b), the cell was quickly placed in a second chamber containing relaxing solution (this explains the mechanical artifact during the transition from pCa 3.88 to pCa 7.5) in order to acquire resting force. See details in the main text (methods). Inserted marks in figures a and b: a. example of a short period of unloaded isotonic shortening and re-stretch to the initial length. b. maximum active isometric force corresponding to the shortening/re-stretch cycle marked with a. c. examples of force redevelopment after shortening/re-stretch cycles. These force redevelopment segments were fitted by a single-exponential function in order to obtain the rate constant of force redevelopment (k_{TR}). **c** An isometric held skinned cardiomyocyte before and after completing a full set of experiments as shown in Fig. 3, including 1 hour of Mt-II exposition (50 µg/mL)

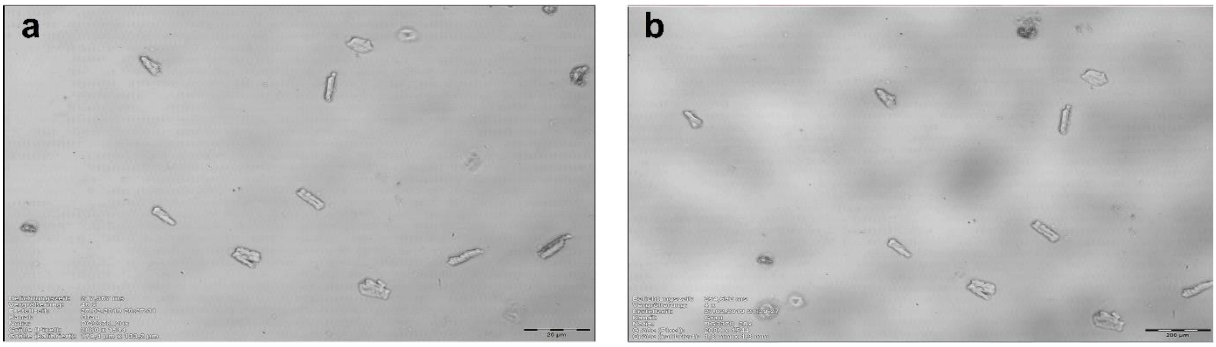

Fig. S2 The myosin inhibitor AmBleb avoided hypercontraction of intact cardiomyocytes exposed to Mt-II. Intact cardiomyocytes were shortly incubated with 50 µM AmBleb in HEPES solution and thereafter exposed to 50 µg/mL Mt-II. Cells did not show hypercontraction after 60 min (a) or 120 min (b) continuous Mt-II exposition. Due to a minimal displacement of the field of view most but not all cells shown in a are also shown in b
